# Applications of the experience sampling method (ESM) in paediatric healthcare: a systematic review

**DOI:** 10.1038/s41390-023-02918-2

**Published:** 2023-12-07

**Authors:** Marije van Dalen, Anne Snijders, Evelien Dietvorst, Katrien Bracké, Sanne L. Nijhof, Loes Keijsers, Manon H. J. Hillegers, Jeroen S. Legerstee

**Affiliations:** 1 Department of Child and Adolescent Psychiatry/Psychology, Erasmus MC Sophia Children’s Hospital, Rotterdam, The Netherlands; 2 Department of Radiology and Nuclear Medicine, Erasmus MC, Rotterdam, The Netherlands; 3 Department of Paediatrics, Wilhelmina Children’s Hospital, University Medical Centre Utrecht, Utrecht University, Utrecht, The Netherlands; 4 Department of Psychology, Education and Child Studies, Erasmus University Rotterdam, Rotterdam, The Netherlands

## Abstract

**Background:**

With the Experience Sampling Method (ESM) participants are asked to provide self-reports of their symptoms, feelings, thoughts and behaviours in daily life. This preregistered systematic review assessed how ESM is being used to monitor emotional well-being, somatic health, fatigue and pain in children and adolescents with a chronic somatic illness.

**Methods:**

Databases were searched from inception. Studies were selected if they included children or adolescents aged 0–25 years with a chronic somatic illness and used ESM focussing on mental health or psychosocial wellbeing, biopsychosocial factors and/or somatic health. Two reviewers extracted data of the final 47 papers, describing 48 studies.

**Results:**

Most studies evaluated what factors influence medical or psychological symptoms and how symptoms influence each other. Another common purpose was to study the feasibility of ESM or ESM as part of an app or intervention. Study methods were heterogeneous and most studies lack adequate reporting of ESM applications and results.

**Conclusions:**

While ESM holds great potential for providing results and feedback to patients and caregivers, little use is being made of this option. Future studies should consider what they report in their studies, conduct a priori power analyses and how ESM can be embedded in clinical practice.

**Impact:**

- While ESM has many clinical applications, it is currently mostly used for research purposes.
- Current studies using ESM are heterogeneous and lack consistent, high-quality reporting.
- There is great potential in ESM for providing patients and parents with personalised feedback.

## Introduction

To ensure that paediatric healthcare professionals adequately support the health and well-being of children and adolescents, it is important that they gain and provide insight into both the physical and mental well-being of their patients. For instance, it may help to better understand how mental and somatic health problems/symptoms are related and interact.^1^ Furthermore, by gaining insight into both the physical and mental well-being of patients, treatment and functional outcomes can be improved^2^ and optimal care using a holistic perspective can be provided.

Historically, healthcare providers have attempted to gain insight into well-being through (retrospective) paper-and-pencil questionnaires, but there are several disadvantages related to this method of data-collection. For instance, questionnaires are affected by recall bias^3^ and they do not enable scholars and clinicians to efficiently examine the context in which the investigated feelings, thoughts or behaviour take place in real-time.^4^

With the rise of technological possibilities in recent years, the number of studies using the Experience Sampling Method (ESM), also called Ecological Momentary Assessment (EMA) or Ambulatory Assessment (AA), have increased both in scientific studies and in clinical practice.^5,6^ In an ESM study, participants report on their thoughts, feelings, symptoms and/or behaviour in their daily life,^7^ typically during multiple (random) times per day for several days or weeks.^3^ Questions may include: Where are you right now? Do you feel tired right now? and Are you alone? The intensive longitudinal data resulting from this data-collection method, may enable both researchers and clinicians^4^ to answer questions on the dynamics of psychological, behavioural and/or medical processes as they occur.^8^

Experience Sampling may have several benefits. First, as ESM is highly suited for inquiring how participants feel, behave and think in the actual context,^3^ it allows researchers and clinicians to relate symptoms, mental well-being and behaviour to contextual factors, such as someone’s whereabouts or their company. Second, ESM may be beneficial for investigating specific age groups such as adolescents.^6^ As adolescents spent on average up to 3 h and 45 min per day on their smartphone,^9^ using applications on their smartphone (ESM apps) may be a convenient way to reach this age group and gather data at different moments during their daily lives. Third, ESM apps provide clinicians and researchers with the ability to provide personalised feedback to their patients.^10^ Or, the ESM apps may provide direct feedback to participants to enable self-monitoring to alleviate symptoms of anxiety or depression.^11,12^

While ESM is increasingly popular in the field of (clinical) psychology and psychiatry,^13,14^ it is also being used in children and adolescents with a chronic somatic illness.^15,16^ For instance, ESM has been combined with Bluetooth sensors on asthma inhalers^17^ or with data from blood glucose meters in adolescents with diabetes.^18^ Since the use of ESM in a paediatric patient population with a chronic somatic illness might have important clinical implications, it is crucial to have an overview on the use of ESM in this particular population. Therefore, this preregistered systematic review aimed to provide an overview of how ESM is used in paediatric healthcare and research. Our main research question was: In which way is ESM used to monitor emotional well-being, somatic health, fatigue and pain of children and adolescents with a chronic somatic illness? More specifically, we sought to answer the following questions: (1) To what purpose do studies deploy ESM? (2) In what way is ESM deployed (i.e., on what device, with which frequency and how long)? and (3) What is the quality of the ESM data and the reporting of ESM data?

## Methods

This article was written in accordance with the Preferred Reporting Items for Systematic Reviews and Meta-Analyses (PRISMA) statement^19^ and the AMSTAR 2 checklist,^20^ and was registered prospectively in the international prospective register of systematic reviews, PROSPERO, registration number CRD42022268954.

### Search strategy

A broad search focusing on the use of ESM in children and adolescents with a chronic somatic illness was conducted by a research librarian from the Erasmus Medical Centre. The search was first conducted on the 19th of July 2021 and updated on the 21st of July 2022. The following databases were searched from inception; Embase, Medline ALL, Web of Science Core Collection, Cochrane Central Register of Controlled Trials and Google Scholar. The terms included in the search were related to Experience Sampling Method, Ecological Momentary Assessment, children, adolescents and paediatrics. The full search can be found in the Supplementary Materials (S1).

### Eligibility criteria

Peer-reviewed studies were eligible if they included children and adolescents (0–25 years of age) with a chronic somatic illness. Chronic somatic illnesses were defined by one or more of the following characteristics: (a) the condition was permanent, (b) left residual disability, (c) was caused by nonreversible pathological alteration, (d) required special training of the patient for rehabilitation or (e) may be expected to require a long period of supervision, observation or care.^21^ In addition, studies were only included if they used ESM or EMA and collected data regarding; (a) mental or psychosocial wellbeing (e.g., affective wellbeing, anxiety, happiness, social functioning, school performance), (b) (psychosomatic) symptoms (e.g., fatigue or pain) or (c) somatic health (e.g., medication use, disease activity). Studies were excluded if they (a) reported no original data (e.g., case reports, conference abstracts, *n* = 1 studies or systematic reviews), (b) used daily dairies or had less than two measurements a day, as these were not deemed to be prototypical ESM^22^ or (c) the article was not written in English. When multiple papers from the same trial were retrieved, only the earliest paper was included in the review.

### Study selection

Studies were selected if they met the inclusion criteria. Two rounds were used to screen the title and abstract. Four reviewers (MA, KB, ED and AS) independently assessed the title and abstract of the articles retrieved in the first search in 2021. The average interrater agreement was 96.82%. Two reviewers (MvD and AS) independently assessed the title and abstract of the papers retrieved in the updated search in July 2022. The average interrater agreement was 94.88%. Subsequently, three reviewers independently assessed the full text articles for eligibility (KB, MvD and AS). The average interrater agreement was 87.41%. In all rounds, consensus was used to resolve discrepancies.

### Data extraction

Two researchers (MvD and AS) performed data-extraction of selected articles. Both researchers extracted data from 50% of selected articles and double-checked the data-extraction for the other 50%. The following information was extracted: general information about the sample (i.e., age, sex, sample size and medical diagnosis), as well as information about the ESM method (e.g., device used, prompt design, duration, ESM intervals, number of prompts, items per assessment and questionnaires) and ESM quality (e.g., compliance rate, timeframe for responding, user experience, reliability).

### Quality and risk of bias

Van Roekel et al. published a checklist for good practices when designing and reporting on ambulatory assessment, which was used for quality and risk of bias assessment. This checklist focusses on participants, procedure (including technology, design of study, participant inclusion and monitoring protocol and compliance) and materials. The checklist was used to assess the quality and risk of bias, with each item rated as 1, 0.5 or 0, or cannot determine/not applicable. Scores were converted to percentages. Papers rated >80% were considered good quality, 60–80% was considered fair quality and <60% was considered poor quality. Quality assessment was done by two reviewers (MvD and AS).

### Data synthesis

Summary statistics were created for the average sample size, sex ratio and average age. When means and standard deviations were not available in the original paper, medians were transformed to means and standard deviations as described by Shi et al.^23^ The final data extraction sheets, reasons for exclusion of full text articles and the quality and risk of bias assessment are available on the Open Science Framework (OSF): https://tinyurl.com/2p8w35ps.

## Results

### Study selection

**Fig. 1:**
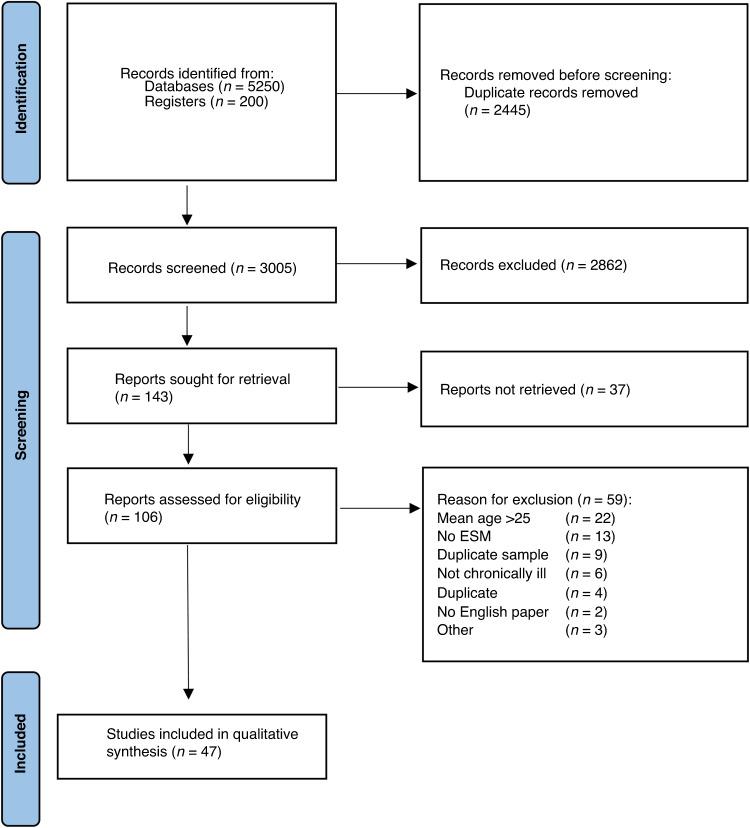
Flow chart of the selection process. Each box represents a step in the literature screening process. The left column represents the number of papers screened at each step, and the right side states the number of documents excluded and the reasons for exclusion.

The literature search yielded 3005 unique records, of which 2862 were excluded based on the title and abstract. Subsequently, 143 records were retrieved for full-text screening, of which 47 were included in the current systematic review. The complete flowchart is shown in Fig. 1. These 47 papers described 48 studies, with a total of 1726 participants. One paper did not report a sample size.^24^ The mean sample size per study was 36.72 participants (range 10 to 88). The weighted mean age of all participants was 14.65 (SD = 2.24) and 23.76% was male. The most common diagnoses studied were asthma (*n* = 9), overweight and/or obesity (*n* = 9) and type 1 diabetes (*n* = 7).

### Purpose of ESM studies

Results of the selected studies are shown in Table 1. The most common purpose of using ESM in children with a chronic illness was to understand what factors influence symptoms and how symptoms influence each other (*n* = 25 studies). These factors may be external, such as relating weather conditions to headaches,^25^ or internal, such as studying the relationship between sleep, pain and daily functioning.^26^ Other common purposes were to study the feasibility of using ESM within a certain patient group (*n* = 9 studies), using ESM to study the feasibility of using an app or intervention for a specific patient group (*n* = 8) or using ESM to study how symptoms of a disease fluctuate over time (*n* = 6). Less common purposes were to study medication adherence (*n* = 5), study social functioning within the context of chronic illness (*n* = 3) or creating self-awareness in participants (*n* = 1).

**Table 1:** Purpose of included studies.

References	Purpose	Sample				Primary outcome measure	Healthcare provider involvement
		Condition	*N*	% male	Age (M, SD)		
Björling & Singh (2017)^51^	Understand fluctuations over time, Understand how symptoms influence each other	Headache	31	0	16.00 (0.97)^a^	Stress	No
Borus et al. (2013)^36^	Study medication adherence	Type 1 diabetes	40	47	16.60 (1.50)	Compliance with glucose monitoring schedules	No
Bray et al. (2010)^52^	Determine the feasibility of using ESM	Neuromuscular disorders	10	100	12.10 (2.50)	Reliability and validity of ESM measures	No
Bray et al. (2017)^53^	Determine the feasibility of using ESM	Duchenne muscular dystrophy	35	100	12.50 (2.80)	Validity of ESM and paper-pencil health-related quality of life	No
Bromberg et al. (2016)^26^	Understand fluctuations over time, Understand how symptoms influence each other	Juvenile idiopathic arthritis	59	26	13.30 (2.80)	Sleep, pain and functional somatic limitations	No
Bui et al. (2020)^70^	Determine the feasibility of an app or intervention	Asthma	20	NR	13.00 (NR)	Unclear; exploratory study	No
Campbell et al. (2006)^71^	Understand how symptoms influence each other	Asthma	53	49	23.00 (2.90)	Airflow obstruction	No
Connelly et al. (2010)^25^	Understand how symptoms influence each other	Headache	25	16	12.34 (2.93)	Headache episodes	No
Connelly & Boorigie (2021)^40^	Determine the feasibility of an app or intervention	Migraine	30	16.7	14.00 (2.10)	Feasibility of data monitoring strategy	No
Cushing et al. (2019)^32^	Determine the feasibility of using ESM	Abdominal pain	34	23.3	13.30 (2.74)	Feasibility; adherence to wearing accelerometer and ESM reports	Yes: clinical team reviews study feedback with child and caregiver during follow-up visit
Cushing et al. (2021)^72^	Understand how symptoms influence each other	Abdominal pain	71	25.4	13.34 (2.67)	Pain severity	No
Dougherty et al. (2022)^35^	Understand how symptoms influence each other	Overweight/obesity	40	47	11.28 (1.91)	Interpersonal stress	No
Dunton et al. (2016)^73^	Understand how symptoms influence each other, Determine the feasibility of using ESM	Asthma	20	54	14.60 (1.70)	Feasibility, compliance and validity of ESM measures	No
Egbert et al. (2020)^45^	Understand how symptoms influence each other	Overweight/ obesity	38	NR	11.16 (1.94)	Loss of control eating/overeating	No
Egbert et al. (2022)^47^ *study 1*	Understand how symptoms influence each other	Overweight/ obesity	36	36	10.61 (1.46)	Loss of control eating	No
Egbert et al. (2022)^47^ *study 2*	Understand how symptoms influence each other	Overweight/ obesity	30	0	14.89 (1.55)	Loss of control eating	No
Feller et al. (2021)^54^	Understand how symptoms influence each other	22Q11DS	37	57	18.32 (4.46)	Psychotic experiences	No
Feller et al. (2022)^42^	Study social functioning within the context of illness	22Q11DS	33	58	19.19 (4.67)	Social functioning	No
Gevonden et al. (2015)^57^	Understand how symptoms influence each other	Severe hearing impairment	15	20	26.50 (2.11)	Social stress	No
Ghriwati et al. (2020)^27^	Understand how symptoms influence each other	Asthma	59	69.5	9.56 (1.53)	Lung functioning	No
Glista et al. (2021)^74^	Determine the feasibility of using ESM	Hearing aids	29	NR	12.14 (2.80)	Adherence to ESM protocol	No
Goldschmidt et al. (2018)^41^	Understand fluctuations over time	Overweight/ obesity	40	45	11.20 (1.90)	Loss of control eating/overeating	No
Hao et al. (2022)^17^	Understand how symptoms influence each other	Asthma	40	55	12.00 (NR)	Lung functioning and inhaler use	No
Heathcote et al. (2022)^33^	Determine the feasibility of using ESM	Childhood cancer survivors	30	50	17.60 (NR)	Feasibility, acceptability and validity of ESM	No
Helgeson et al. (2009)^18^	Study social functioning within the context of illness	Type 1 diabetes	76	50	14.54 (.95)	Depressive symptoms, self-care behaviour and metabolic control	No
Jessup et al. (2017)^28^	Study social functioning within the context of illness	Visual impairment	12	41.67	NR	Social inclusion	No
Kichline et al. (2019)^75^	Understand fluctuations over time, Understand how symptoms influence each other	Chronic abdominal pain	71	25.4	13.34 (2.67)	Physical activity levels	No
Kolmodin MacDonell et al. (2016)^76^	Determine the feasibility of an app or intervention	Asthma	49	25.59	22.44 (3.71)	Feasibility and acceptability of medication-adherence intervention	Unclear
Kubiak et al. (2018)^77^	Understand how symptoms influence each other	Obesity	16	0	15.50 (1.40)	Emotional eating	No
Lee et al. (2020)^43^	Determine the feasibility of using ESM	Juvenile idiopathic arthritis	14	36	12.14 (3.30)^a^	Feasibility of different ESM protocols	No
MacDonell et al. (2012)^46^	Determine the feasibility of using ESM	Asthma	16	43.75	19.75 (1.77)	Feasibility of ESM protocol	No
Miadich et al. (2018)^48^	Understand how symptoms influence each other	Asthma	54	68.5	9.52 (1.51)	Sleep quality	No
Mulvaney et al. (2012)^56^	Determine the feasibility of using ESM	Type 1 diabetes	50	50.1	15.11 (1.60)	Feasibility and adherence to ESM	No
Mulvaney et al. (2018)^38^	Determine the feasibility of an app or intervention, Creating self-awareness	Type 1 diabetes	30	48.39	15.42 (1.54)	Feasibility and utility of ESM	No
Nap-van der Vlist (2021)^31^	Determine the feasibility of an app or intervention	Illnesses associated with fatigue	57	16	16.20 (1.60)	Feasibility and usefulness of app	Yes: choosing ESM content and discussing personalised report
Psihogios et al. (2021)^30^	Study medication adherence, Determine the feasibility of an app or intervention	Acute lymphoblastic leukaemia	18	77.80	17.94 (2.31)	Feasibility and adherence to ESM	Yes: summary of adherence was discussed during clinic visit
Rancourt et al. (2015)^49^	Understand how symptoms influence each other	Obesity	46	0	19.02 (2.61)	Weight-related thoughts and behaviour	No
Rofey et al. (2010)^78^	Understand how symptoms influence each other	Obesity	20	0	NR	Feasibility of ESM	No
Schurman & Friesen (2015)^50^	Understand how symptoms influence each other	Chronic abdominal pain	13	23.08	13.50 (2.40)	Abdominal pain	No
Shapira et al. (2020)^58^	Understand fluctuations over time, Understand how symptoms influence each other	Type 1 diabetes	32	44	16.60 (1.40)	Adherence to blood glucose checks, blood glucose levels and glucose variability	No
Smith et al. (2021)^55^	Understand how symptoms influence each other	Obesity	38	58.4	15.06 (1.39)	Physical activity levels	No
Stinson et al. (2014)^24^	Understand fluctuations over time, Understand how symptoms influence each other	Juvenile idiopathic arthritis	NR	NR	NR	Pain intensity	No
Sweenie et al. (2022)^37^	Study medication adherence	Asthma	25	48	14.70 (1.68)	Adherence to asthma medication	No
Tasian et al. (2019)^39^	Understand how symptoms influence each other	Nephrolithiasis	25	40	16.00 (0.97)^a^	Daily water intake	No
Teufel et al. (2018)^29^	Determine the feasibility of an app or intervention	Asthma	14	36	CD	Feasibility of ESM	Yes: developing ESM questions, data available for real-time review in web-based portal
Valrie et al. (2019)^34^	Understand how symptoms influence each other	Sickle cell disease	88	41	11.66 (2.99)	Sleep quality, duration, efficiency and latency	No
Warnick et al. (2020)^79^	Study medication adherence, Determine the feasibility of an app or intervention	Type 1 diabetes	62	56.5%	16.40 (3.00)	Validity of ESM and adherence to blood glucose monitoring	No
Zhang et al. (2022)^15^	Study medication adherence	Type 1 diabetes	45	47%	13.30 (1.70)	Missed self-management (i.e., monitoring glucose, administering insulin)	No

^a^Estimated using the method described by Shi et al.^23^.

*CD* Cannot determine, *NR* Not reported.

Studies used different primary outcome measures. Most studies (*n* = 17) looked at the feasibility of ESM, either as part of an app or intervention or as a stand-alone methodology. Other studies (*n* = 10) looked at medical outcomes such as pain intensity^24^ or lung functioning,^27^ or at psychological outcomes (*n* = 7) such as social inclusion^28^ and depressive symptoms.^18^ A complete overview is shown in Table 1.

There were no studies using ESM as independent application for patient self-monitoring. Four out of 48 studies reported involving a healthcare provider in the ESM protocol and results.^29–32^ In two of these studies, the healthcare providers were involved in choosing the content of the micro-questionnaires. Three of the four studies reported that the healthcare provider discussed the results of the ESM with the patient and caregiver. One study reported that the data was available for the healthcare professional, but does not mention the data being discussed with the patient. The remaining studies do not mention the involvement of the healthcare provider, except for Heathcote et al.,^33^ where a visit to the outpatient clinic was part of protocol. However, this visit did not include discussing ESM results.

### Characteristics of ESM

See Table 2 for an overview of technical and design characteristics of the ESM.

**Table 2:** Overview of ESM uses in different studies.

References	Condition	Controls	Device	Prompt design	Duration	Number of daily prompts	Number of items	Compliance	Incentives
Björling & Singh (2017)^51^ ^a^	Headache	No	Palm pilot M500	CD, random	21 days	7	5	72%	$75
Borus et al. (2013)^36^	Type 1 diabetes	No	Palm Tungsten E2	Fixed event-contingent	14 days	4	NR	63%	$100 for >75% compliance
Bray et al. (2010)^52^	Neuromuscular disorders	No	Palm Z22	Random signal-contingent	7 days	8	19	79%	NR
Bray et al. (2017)^53^	Duchenne muscular dystrophy	No	Palm Z22	Random signal-contingent	7 days	8	19	70%	NR
Bromberg et al. (2016)^26^	Juvenile idiopathic arthritis	No	Smartphone	Fixed signal-contingent	1 month	3	9–14	66%	Dependent on compliance
Bui et al. (2020)^70^	Asthma	No	Smartphone + wearable	NR	1 week	NR	NR	NR	CD
Campbell et al. (2006)^71^	Asthma	No	Paper & pencil + wearable	Interval-contingent	13–16 h	13-16	NR	NR	NR
Connelly et al. (2010)^25^	Headache	No	Palm device	Time-contingent	14 days	3	14	84%	Dependent on compliance
Connelly & Boorigie (2021)^40^	Migraine	No	Smartphone + wearable	Time-contingent	28 days	4	11–12	68.9%	Dependent on compliance
Cushing et al. (2019)^32^	Abdominal pain	No	Smartphone + wearable	Time-contingent	14 days	4	45–61	76.3%	Dependent on compliance
Cushing et al. (2021)^72^	Abdominal pain	No	Smartphone	NR	14 days	4	NR	73%	CD
Dougherty et al. (2022)^35^	Overweight/obesity	No	Smartphone	Random signal-contingent, interval-contingent & event-contingent	16 days	4–6	10	56% (signal-contingent), 68.6% (interval-contingent)	$100–$150
Dunton et al. (2016)^73^	Asthma	No	Smartphone + wearable	Random signal-contingent & event-contingent	7 days	CD	NR	50.1%	$100
Egbert et al. (2020)^45^	Overweight/ obesity	No	NR	Random signal-contingent, event-contingent & interval-contingent	15 days	NR	20	NR	NR
Egbert et al. (2022)^47^ *study 1*	Overweight/ obesity	No	Mobile phone + phone calls	NR	4 days	3	>4	74%	NR
Egbert et al. (2022)^47^ *study 2*	Overweight/ obesity	No	Palm Pilot PDA	Signal-contingent & event-contingent	15 days	3-5	NR	69% (signal-contingent)	NR
Feller et al. (2021)^54^	22Q11DS	49 healthy controls	Smartphone	Semi-random signal-contingent	6 days	8	33-36	NR	NR
Feller et al. (2022)^42^	22Q11DS	44 healthy controls	Smartphone	Semi-random signal-contingent	6 days	8	33-38	NR	€90,- or 100 Fr.
Gevonden et al. (2015)^57^	Severe hearing impairment	18 healthy controls	PsyMate	Semi-random signal-contingent	8 days	10	NR	NR	€50,-
Ghriwati et al. (2020)^27^	Asthma	No	Smartphone + wearable	Fixed signal-contingent	14 days	2	NR	NR	$25-$50
Glista et al. (2021)^74^	Hearing aids	No	Asus Zenpad 7 tablet	Event-contingent	1 week	2	NR	82.4%	CD
Goldschmidt et al. (2018)^41^	Overweight/ obesity	No	Smartphone	Semi-random signal-contingent, event-contingent & interval-contingent	15 days	3-5	NR	23.3%-67.6%	$50-$100
Hao et al. (2022)^17^	Asthma	No	Smartphone + wearable	Random signal-contingent, interval-contingent & event-contingent	14 days	NR	NR	NR	NR
Heathcote et al. (2022)^33^	Childhood cancer survivors	No	Smartphone	Semi-random signal-contingent	11 days	3	NR	83%	Dependent on compliance
Helgeson et al. (2009)^18^	Type 1 diabetes	No	Palm pilot + blood glucose meter	Fixed signal-contingent	4 days	6-9	NR	CD	$100
Jessup et al. (2017)^28^	Visual impairment	No	Smartphone	Random signal-contingent	1 week	7	NR	69%	NR
Kichline et al. (2019)^75^	Chronic abdominal pain	No	Smartphone + wearable	Fixed signal-contingent	14 days	4	4	73%	$40
Kolmodin MacDonell et al. (2016)^76^	Asthma	No	Smartphone	Random signal-contingent	7 days + 7 days	3	NR	NR	$200
Kubiak et al. (2018)^77^	Obesity	No	Palm Tungsten E2	Random signal-contingent & event-contingent	7 days	4	NR	CD	CD
Lee et al. (2020)^43^	Juvenile idiopathic arthritis	No	iPad	NR	8 weeks	1-2	NR	37.8–63%	NR
MacDonell et al. (2012)^46^	Asthma	No	Phone	Fixed signal-contingent & event-contingent	14 days	1-2	NR	78.5% (signal-contingent)	$100 + raffle
Miadich et al. (2018)^48^	Asthma	No	Smartphone	Fixed signal-contingent	2 weeks	2	1–6	CD	NR
Mulvaney et al. (2012)^56^	Type 1 diabetes	46 type 1 diabetes, without ESM	Phone calls	Semi-random signal-contingent	20 days	2	4	CD	NR
Mulvaney et al. (2018)^38^	Type 1 diabetes	14 type 1 diabetes, without ESM	Smartphone	Fixed signal-contingent & event-contingent	30 days	4	NR	64.17%	$60–$100
Nap-van der Vlist (2021)^31^	Illnesses associated with fatigue	No	Smartphone	Fixed signal-contingent	10–67 days	5	<27	42%	NR
Psihogios et al. (2021)^30^	Acute lymphoblastic leukaemia	No	Smartphone + wearable	Fixed signal-contingent & event-contingent	28 days	3–4	2–9	79.5%-88.9%	Dependent on compliance
Rancourt et al. (2015)^49^	Obesity	No	Royal Brand PDA	Random signal-contingent	5 days	6	8	NR	Study credits
Rofey et al. (2010)^78^	Obesity	No	Phone calls + wearable	Random signal-contingent	NR	2-4	NR	64.2%	NR
Schurman & Friesen (2015)^50^	Chronic abdominal pain	No	Palm device	Fixed signal-contingent	14 days	3	CD	86%	NR
Shapira et al. (2020)^58^	Type 1 diabetes	No	Palm Tungsten E2	Fixed signal-contingent	2 weeks	4	12	72% (median)	NR
Smith et al. (2021)^55^	Obesity	39 non-obese siblings	Cell phone + wearable	Interval-contingent	7 days	4-7	5	95.0%	CD
Stinson et al. (2014)^24^	Juvenile idiopathic arthritis	No	Palm Tungsten W PDA	Fixed signal-contingent	14 days	3	CD	78%	NR
Sweenie et al. (2022)^37^	Asthma	No	Phone + wearable	Fixed signal-contingent	21 days	4	12	67.5%	$25 or $40
Tasian et al. (2019)^39^ ^a^	Nephrolithiasis	No	Phone	Random signal-contingent	7 days	4	CD	85%	NR
Teufel et al. (2018)^29^	Asthma	No	Smartphone + wearable	Signal-contingent	2 months	1	8	20%	$50 or $200
Valrie et al. (2019)^34^	Sickle cell disease	No	Smartphone + wearable	Semi-fixed signal-contingent	4 weeks	2	6-10	81.87%	$20 or $60
Warnick et al. (2020)^79^	Type 1 diabetes	No	Smartphone	Fixed signal-contingent	10 days	3	4	43.80%	$10
Zhang et al. (2022)^15^	Type 1 diabetes	No	Smartphone + blood glucose meter	Fixed signal-contingent	30 days	CD	CD	NR	NR

^a^Estimated using the method described by Shi et al.^23^

*CD* Cannot determine, *NR* Not reported.

#### Software/Devices

The majority of studies (*n* = 24) reported using either a loaned or owned smartphone to disseminate the ESM. An additional five studies did not provide clarity on whether they used a smartphone or other (mobile) phone. Some other studies reported using a personal digital assistant. Most of these studies used a Palm device (*n* = 11), but Royal Brand (*n* = 1) has also been used. As not all studies reported their time frame of data collection, there was not enough information to determine whether these were primarily older studies. Fifteen studies combined the ESM device with a wearable, such as blood glucose meters, Bluetooth asthma inhaler caps or accelerometers. The majority of the studies using a wearable combined this with ESM via a smartphone application.

#### Sampling scheme

The duration of the ESM studies ranged from 13 h to two months, with the majority of the studies (70.83%) using ESM for 14 days or less (median = 14, IQR 7; 15.75). The number of prompts per day ranged from 1 to 16 prompts^a^, with a mean of 4.3 prompts per day and a mean of 54.37 prompts over the course of the study (range 12 to 147). Ten studies had different numbers of prompts for different days, often participants received more prompts during the weekend than during weekdays.

#### Micro-questionnaires

Most studies included around 10–20 items, with a range of 4 to 61 items. However, almost half of the studies (*n* = 24) did not report the number of items they included in their ESM prompts.

#### Compliance and incentives

To ensure sufficient compliance (i.e., number of answered questionnaires), half of the studies (47.92%) motivated participants with financial means or through study credits (2.08%). Most studies with a financial incentive used different incentives based on the compliance rate or completed research visit. For instance, participants received money at the start of the study and at the final research visit.^29,34,35^ Other studies gave participants money if the compliance was at least 70%,^36^ 75%,^37^ 80%,^32,38^ 85%^39^ or 90%.^40^ A third approach was to add a small amount of money (e.g., $0.25 to $2.50^25,26,33,41^) to the total incentive for each ESM measure completed. Nineteen studies did not report whether their participants received an incentive for participating.

#### Personalised feedback

Eight studies reported giving participants insight into their ESM results. This feedback was provided by a healthcare provider^32^ or researcher,^18^ but most often through a personalised report,^15,30,31,38,42,43^ which was sometimes discussed by a healthcare provider f.e.^31^

### Quality of ESM data and studies

The quality of the ESM studies was determined using the checklist for good practice when reporting on ambulatory assessment.^6^ The results are shown in Table 3. Overall, 27 studies (56.25%) were deemed to be of poor quality. The remaining 21 studies (43.75%) were of fair quality. No studies were of good quality.

**Table 3:**
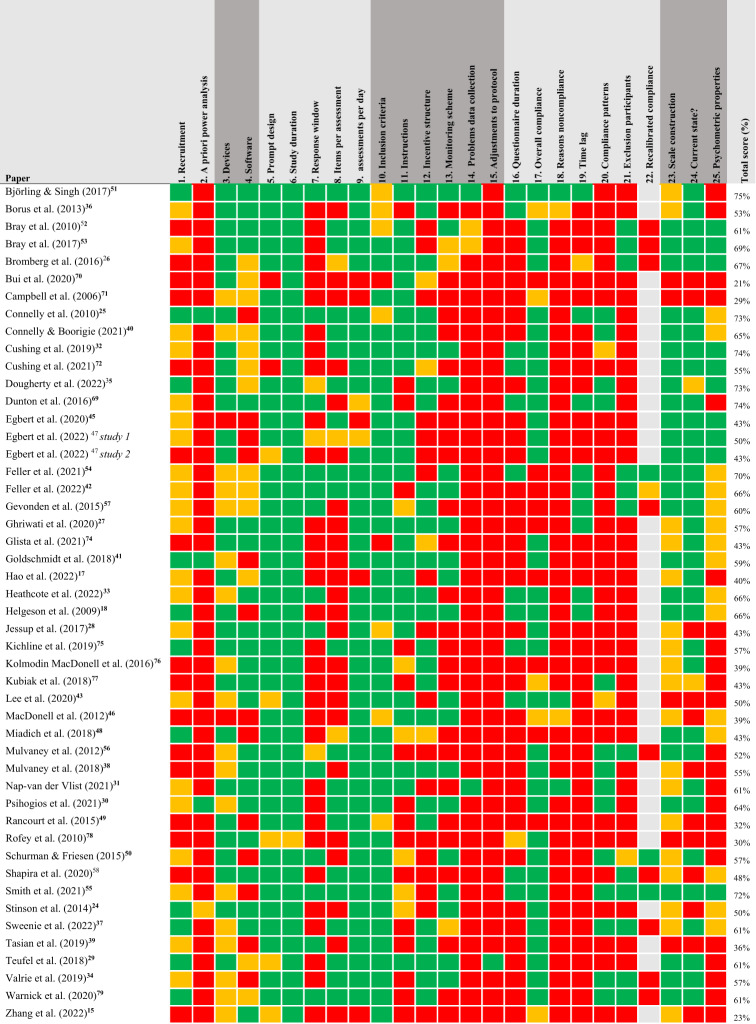
Quality assessment of included studies.

green = 1 point, yellow = 0.5 point, red = 0 points or cannot determine, grey = not applicable.

Checklist questions: 1. Report on specific recruitment methods (e.g., effective strategies to ensure school participation); 2. A priori power analysis, based on sample size, number of assessments, and smallest effect size of interest; 3. Devices (including versions), when relevant (e.g., % of participants who use an IOS vs. Android smartphone); 4. Software; 5. Prompt design (i.e., signal-contingent, interval-contingent, event-contingent; random vs. fixed intervals); 6. Study duration; 7. Response window (i.e., how much time do the participants have to complete a questionnaire?); 8. Total number of items per assessment; 9. Number of assessments per day; 10. Exclusion or inclusion criteria; 11. The instructions that were given to participants; 12. Incentive structure (i.e., what compensation was provided to participants?); 13. Monitoring scheme (i.e., if, how many, and when automatic reminders were sent; whether and under which circumstances participants were contacted, which messages were sent); 14. Any problems during data collection; 15. Adjustments to protocol; 16. Questionnaire duration (i.e., average questionnaire duration as well as measures of variability, e.g., SD, CI).; 17. Overall compliance (i.e., average number and percentage of completed assessments, including measure of variability such as SD, or a plot visualizing this variability); 18. Reasons for noncompliance (e.g., technical problems, response window passed, illness reported); 19. Time lag between prompt and completed assessment (i.e., is compliance based on assessments completed within a certain time window or on all assessments?); 20. Patterns of noncompliance and missing data; 21. Were participants excluded for analyses based on compliance rates? If so, what cut-off was used?; 22. If relevant: Compliance after exclusion of participants; 23. Scale construction and transformation (including centering); 24. Are participants asked about their current state (in-the-moment) or about the past hour(s)/day?; 25. Psychometric properties of scales (e.g., within-person reliability).

#### Power

Table 1 shows most studies had small sample sizes (M = 36.72 participants), with sample sizes ranging from 10 to 88 participants. Twelve studies (25.00%) included 20 participants or less. Notably, only four studies performed an a priori power analysis, using computer programs to generate either minimum sample sizes with moderate regression coefficients^24,25,30^ or multi-level Monte Carlo simulations.^41^ One study based their sample size on the minimum recommended sample size for multilevel designs by Maas and Hox,^44^ instead of performing a formal statistical power analysis.^40^ However, eight studies were feasibility studies. A power analysis may not be applicable to those studies.

#### Reporting

Several studies lacked sufficient details in the methods section to replicate the study. For instance, two studies did not report on the software nor the devices used to gather the ESM data.^45,46^ An additional 11 studies reported on the devices used during the study but did not report which software was used.^18,25,34,39,41,46–50^

Most studies (95.83%) described the prompt design, reporting on both intervals (e.g., random intervals or fixed intervals) and/or prompt contingent (e.g., signal-contingent or event-contingent). Sixteen out of 48 studies reported on the response window available for participants to complete the ESM after being given the prompt.

About half of the studies (*n* = 22) also reported a monitoring scheme, detailing when and how many reminders participants received to ensure compliance. Most studies used automated reminders,^29^ contacted the participant at least once a week^24,34,41–43,51–54^ or contacted participants when compliance rates declined.^17,26,35,55^ A few studies combined automated reminders with contacting participants.^30,38^

Four studies reported problems during data collection (mostly related to technical issues). None of these studies reported subsequent adjustments to protocol.^31,51–53^ The other studies (*n* = 44) reported no problems or technical issues during data collection. It is unclear whether this indicates that there were no problems or whether problems were not reported.

#### Compliance

The majority of the studies (39 out of 48/81%) reported the overall compliance. Table 2 shows that the overall compliances range from 20% to 95% (M = 69.77, SD = 14.87). Some studies (*n* = 13) reported excluding participants based on compliance score. Six studies reported a compliance cut off score. These cut off scores varied between ≥25%,^26,56^ or ≥33%^42,54,57^ or ≥50%.^52^ In addition, two studies omitted ESM data that could not be matched to the data points of a wearable.^55,58^ Of these 13 studies, three studies reported recalibrating the overall compliance after exclusion of participants.^42,55,59^ Other studies (*n* = 10) only included questionnaires completed within a certain time window. The remaining 25 studies did not provide clarity on the overall compliance. Notably, only five studies reported reasons for noncompliance, such as technical problems, illness or missed time windows. Fifteen studies provided some insight into compliance reasons by reporting on (non)compliance patterns. Two studies reported compliance was higher for the morning questionnaires and on the weekend days.^30,50^

#### User experience

Twelve out of 48 studies reported on user experience, typically based on satisfaction with the study procedures and willingness to participate again. Most studies used either a questionnaire or brief interview. Four studies indicated that the majority of participants thought ESM was easy to use and gave a positive recommendation for peers.^31–33,43^

#### Materials

The last three items of the checklist for good practice assess the materials used in the studies. Almost all studies (*n* = 43) reported on scale construction and transformation. Over half of these studies (*n* = 25) also reported on the psychometric properties of the used scales. Five studies did not report on scale construction and transformation, nor on the psychometric properties of the scale. Lastly, thirty-five articles specified whether the participants answered the ESM questions about their current state (in-the-moment) or about the past hour(s).

## Discussion

Whereas ESM is booming in clinical psychology, psychiatry and other fields of study (e.g., communication sciences, organisational psychology),^5,6^ relatively little is known about its application in paediatrics, despite showing promise for the field. Hence, this preregistered systematic review aimed to provide an overview of the application of ESM in paediatrics. More specifically, we aimed to study the purposes of the studies using ESM, the way ESM was deployed and the quality of the ESM studies as well as their reporting. A systematic literature search yielded 47 papers, describing 48 studies.

Almost all studies had an aim that was primarily related to doing research. Most often, the purpose was to investigate what factors influenced medical or psychological symptoms and how symptoms influenced each other. With regards to using ESM to provide personalised feedback, only one study used ESM to create self-awareness in participants and eight studies gave participants insight into their ESM results, of which five without professional guidance or support. Four studies involved the healthcare professional in the ESM methodology or results. Previous ESM applications in psychology provided personalised feedback to participants,^60^ so they may change their behaviour^61^ or to alleviate psychological symptoms.^62^ Similar applications could also be used in paediatrics to benefit both the healthcare provider and the patient or caregiver. For instance, monitoring of symptoms or medication adherence may provide the healthcare provider with useful insights for monitoring wellbeing and treatment adherence,^63^ as well as insight into the influence of contextual factors on the physical and mental well-being. The patient and caregiver may also gain insight and adopt their behaviour according to the feedback provided. Using ESM in this way also aligns with recent developments in paediatrics, such as value-based healthcare and shared decision making.^64^ In addition, by using ESM in routine outcome monitoring, more positive health outcomes and a subsequent reduction in healthcare costs may be realised.^65,66^

In comparison to other fields of study, the field of ESM in paediatrics seems in its infancy, both in terms of the number of studies as in terms of quality indicators. Most existing studies in paediatrics were much smaller than typical ESM studies on adolescents in other fields of study.^6^ In our review, the mean sample size was 36.72 and the average number of assessments was 54.37. Whether this was sufficient is an urgent question, as the topic of how precise estimates from ESM data are is still under investigation by methodologists. However, it may less feasible to recruit large samples of adolescents with a chronic illness into studies compared to large samples of adolescents from the general population. As not all adolescents are diagnosed with a chronic illness, the group of potential participants is much smaller and large study samples may thus be harder to achieve.

With regards to the quality of reporting, ESM research in paediatrics may benefit from developments in other domains. Many of the selected studies lacked sufficient details to replicate work (e.g., reasons for noncompliance or patterns of (non)compliance). Although the study design was often well-described, studies often omitted reporting the response window, the amount of items per assessment or the monitoring scheme. Hence, we recommend that future research make use of the checklist published by van Roekel et al.^6^ for reporting their findings and may also benefit from a strong Open Science Movement with regards to good practices for ESM.^67^

This study also has several strengths and limitations. This is the first overview of how ESM is applied in paediatrics. A particular strength is its preregistered design with a thorough literature search. A first limitation is that the checklist used to assess quality and risk of bias assessment was developed primarily for studies within the field of psychology. Hence, the quality of reporting relating the chronic illness itself was not assessed. A second limitation is that our search was tailored primarily to chronic illness and ESM, but not towards wearables. This review may not provide a complete overview of the application of wearables in paediatrics.

This overview can serve as inspiration for clinicians working with children with a chronic illness. ESM can be embedded in the clinical practice in many ways, for instance by combining ESM with data from wearables (e.g., heart rate monitors or blood glucose meters). Another possibility is to provide feedback to patients and parents either by the healthcare provider themselves, or through personalised reports. In terms of the duration of ESM, this systematic review showed there are many possible durations. Another option that was not highlighted in this review, but has been previously suggested is to incorporate ESM throughout various stages of the treatment,^68^ for instance by starting with a few days ESM at baseline and doing a follow-up period after six weeks.

Future research using ESM for either scientific or clinical purposes can be strengthened by learning from other domains. For instance, studies should conduct a priori power analyses (e.g., using Mplus or PowerAnalysisIL), and items and questionnaires (including branching and dependencies) could be shared through open science, in repositories such as the ESM item repository.^69^ Following guidelines for reporting ESM studies, authors should provide stronger rationales for their sample schemes and frequencies to enable replication and faster progress in paediatric research and practice. In addition, future research should establish whether the use of ESM in clinical practice may lead to a reduction of healthcare costs.

In conclusion, there are many different applications of ESM in paediatrics. Although the reporting of many papers can be improved, these applications may be of inspiration to other researchers and healthcare professionals. Despite the field of ESM in paediatrics being in its infancy, ESM can be embedded into the healthcare process in a myriad of ways. Incorporating ESM into healthcare could also ensure a reduction in healthcare costs by enhancing treatment adherence through personal feedback, or by allowing clinicians to provide early interventions based on ESM responses. However, this should be investigated in future studies.

### Supplementary information

PRISMA 2020 Main Checklist

Supplementary material

## Data Availability

The datasets generated during the current study are available in the OSF repository, https://osf.io/k7z63/?view_only=b479f9ee620f43acaf0242c4aa21486a.
