# Supplementary material for: Applications of the experience sampling method (ESM) in paediatric healthcare: a systematic review
Source: Pediatr Res. 2023 Dec 7;95(4):887–900. doi: 10.1038/s41390-023-02918-2 (PMC10920184; doi:10.1038/s41390-023-02918-2)
Supplement: Supplementary file 2 — Supplementary material [file 41390_2023_2918_MOESM2_ESM.pdf]

Supplementary material – Search strategy

Embase

('ecological momentary assessment'/de OR (momentary-assessment* OR ambulatory-assessment* OR experience-sampling*):ab,ti,kw) **AND** (child/exp OR adolescent/exp OR adolescence/exp OR 'child behavior'/de OR 'child parent relation'/de OR pediatrics/exp OR childhood/exp OR 'child nutrition'/de OR 'infant nutrition'/exp OR 'child welfare'/de OR 'child abuse'/de OR 'child advocacy'/de OR 'child development'/de OR 'child growth'/de OR 'child health'/de OR 'child health care'/exp OR 'child care'/exp OR 'childhood disease'/exp OR 'child death'/de OR 'child psychiatry'/de OR 'child psychology'/de OR 'pediatric ward'/de OR 'pediatric hospital'/de OR 'pediatric anesthesia'/de OR 'pediatric intensive care unit'/de OR 'neonatal intensive care unit'/de OR 'prematurity'/de OR (adolescen* OR preadolescen* OR infan* OR newborn* OR (new NEXT/1 born*) OR baby OR babies OR neonat* OR prematur* OR pre-matur* OR child* OR kid OR kids OR toddler* OR teen* OR boy* OR girl* OR minors OR underag* OR (under NEXT/1 (age* OR aging OR ageing)) OR juvenil* OR youth* OR kindergar* OR puber* OR pubescen* OR prepubescen* OR prepubert* OR pediatric* OR paediatric* OR school* OR preschool* OR highschool* OR suckling* OR PICU OR NICU OR PICUs OR NICUs):ab,ti,kw)

Medline

(Ecological Momentary Assessment/ OR (momentary-assessment* OR ambulatory-assessment* OR experience-sampling*).ab,ti,kf.) **AND** (exp Child/ OR exp Infant/ OR exp Adolescent/ OR exp "Child Behavior"/ OR exp "Parent Child Relations"/ OR exp "Pediatrics"/ OR "Child Nutrition Sciences"/ OR "Infant nutritional physiological phenomena"/ OR exp "Child Welfare"/ OR "Child Development"/ OR exp "Child Health Services"/ OR exp "Child Care"/ OR "Child Rearing"/ OR exp "Child development Disorders, Pervasive"/ OR "Child Psychiatry"/ OR "Child Psychology"/ OR "Hospitals, Pediatric"/ OR exp "Intensive Care Units, Pediatric"/ OR (adolescen* OR infan* OR newborn* OR (new ADJ born*) OR baby OR babies OR neonat* OR prematur* OR pre-matur* OR child* OR kid OR kids OR toddler* OR teen* OR boy* OR girl*

OR minors OR underag* OR (under ADJ1 (age* OR aging OR ageing)) OR juvenil* OR youth* OR kindergar* OR puber* OR pubescen* OR prepubescen* OR prepubert* OR pediatric* OR paediatric* OR school* OR preschool* OR highschool* OR suckling* OR PICU OR NICU OR PICUs OR NICUs).ab,ti,kf.)

Cochrane
(((experience NEXT/1 sampling*) OR (momentary NEXT/1 assessment*) OR (ambulatory NEXT/1 assessment*)):ab,ti,kw) **AND** ((adolescen* OR preadolescen* OR infan* OR newborn* OR (new NEXT/1 born*) OR baby OR babies OR neonat* OR prematur* OR (pre NEXT/1 matur*) OR child* OR kid OR kids OR toddler* OR teen* OR boy* OR girl* OR minors OR underag* OR (under NEXT/1 (age* OR aging OR ageing)) OR juvenil* OR youth* OR kindergar* OR puber* OR pubescen* OR prepubescen* OR prepubert* OR pediatric* OR paediatric* OR school* OR preschool* OR highschool* OR suckling* OR PICU OR NICU OR PICUs OR NICUs):ab,ti,kw)

Web of Science
TS=(((momentary-assessment* OR ambulatory-assessment* OR experience-sampling*)) AND ((adolescen* OR preadolescen* OR infan* OR newborn* OR (new NEAR/1 born*) OR baby OR babies OR neonat* OR prematur* OR pre-matur* OR child* OR kid OR kids OR toddler* OR teen* OR boy* OR girl* OR minors OR underag* OR (under NEAR/1 (age* OR aging OR ageing)) OR juvenil* OR youth* OR kindergar* OR puber* OR pubescen* OR prepubescen* OR prepubert* OR pediatric* OR paediatric* OR school* OR preschool* OR highschool* OR suckling* OR PICU OR NICU OR PICUs OR NICUs)))

Google Scholar
"ecological*assessment"|"experience sampling" child|infant|pediatric|paediatric|adolescent
